# Entire solutions for a reaction–diffusion equation with doubly degenerate nonlinearity

**DOI:** 10.1186/s13662-018-1606-y

**Published:** 2018-04-24

**Authors:** Rui Yan, Xiaocui Li

**Affiliations:** 1 School of Applied Mathematics, Shanxi University of Finance and Economics, Taiyuan, P.R. China; 2 School of Science, Beijing University of Chemical Technology, Beijing, P.R. China

**Keywords:** 35K57, 35B40, Entire solutions, Traveling wave solutions, Doubly degenerate nonlinearity

## Abstract

This paper is concerned with the existence of entire solutions for a reaction–diffusion equation with doubly degenerate nonlinearity. Here the entire solutions are the classical solutions that exist for all $(x,t)\in \mathbb{R}^{2}$. With the aid of the comparison theorem and the sup-sub solutions method, we construct some entire solutions that behave as two opposite traveling front solutions with critical speeds moving towards each other from both sides of *x*-axis and then annihilating. In addition, we apply the existence theorem to a specially doubly degenerate case.

## Introduction

In this paper, we consider the following scalar reaction–diffusion equation:

1.1 $$ u_{t}=u_{xx}+f(u), $$

where *f* satisfies $(A)$ $f\in C^{2} ([0,2] ), f(0)=f(1)=0, f'(0)=f'(1)=0$, $f'(s)>0, f'(1-s)<0$ for small $s>0$, and $f(u)>0$ for $u\in(0,1)$.

From $(A)$, it is easy to see that $u=0, u=1$ are two constant equilibria of (1.1).

In practical applications, traveling wave solutions can well explain oscillations and finite velocity propagation phenomena in nature. Thus, in recent years, the existence and the stability of traveling wave solutions of (1.1) have been extensively studied by many scholars, see [1–5] and the references therein. A traveling wave solution of (1.1) is a solution of the form $u(x,t)=\phi(x+ct)$ satisfying

1.2 $$\begin{aligned} \textstyle\begin{cases} \phi''-c\phi'+f(\phi)=0,\\ \phi(-\infty)=0,\qquad \phi(+\infty)=1, \end{cases}\displaystyle \end{aligned}$$

where $':=\frac{{\mathrm {d}}}{{\mathrm {d}}z}, z=x+ct$ and *c* is the wave speed. Moreover, a monotone and bounded traveling wave solution is called a traveling front solution. In [3], Hou et al. investigated the existence, uniqueness, asymptotic behavior as well as the stability of the traveling front solutions for (1.1).

However, it is not enough to understand the dynamical structure of solutions of reaction–diffusion equations by only considering traveling front solutions. From the dynamical view, the study of entire solutions is essential for a full understanding of the transient dynamics and the structures of the global attractor as mentioned in [6]. For example, the *ω*-limit sets of bounded solutions of (1.1) and global attractor are comprised of entire solutions as mentioned in [6, 7]. In recent years, the existence of entire solutions is widely discussed. Firstly, when *f* satisfies

1.3 $$ f'(0)>0,\qquad f'(1)< 0,\qquad f(u)>0,\qquad f'(u)\leq f'(0),\quad \mbox{for all } u\in(0,1), $$

then (1.1) becomes the famous Fisher–KPP equation (monostable case). For this equation, Hamel and Nadirashvili in [8] proved the existence of entire solutions by the comparison theorem and super-sub estimates, which consists of traveling front solutions and solutions to the diffusion-free equations. Moreover, they also pointed out that the entire solutions to (1.1) depend only on *t* and traveling wave solutions are typical examples of entire solutions, and they showed various entire solutions of (1.1) with (1.3) in their subsequent paper [9]. While for both $f'(0)<0$ and $f'(1)<0$ (bistable case), Yagisita in [10] revealed that the annihilation process is approximated by a backward global solution of (1.1), which is the entire solution. We call this kind of entire solutions annihilating entire solutions, and in the following, without special indication, we use entire solutions to represent annihilating entire solutions for short. For Allen–Cahn equation

$$u_{t}=u_{xx}+u(1-u) (u-a), $$

with $a\in(0,1)$, as a special example in [10], Fukao et al. in [11] gave a proof for the existence of entire solutions by using the explicit expression of the traveling front and the comparison theorem. Later, Guo and Morita in [12] extended the results in [8] and [10] to more general cases including a discrete KPP equation by the super-sub solutions method and the comparison theorem. At the same time, Chen and Guo in [13] proved the existence of entire solutions by showing a different technique which used only one function to construct a pair of deterministic super-sub solutions. Moreover, Chen et al. in [14] also used the method in [13] to construct entire solutions based on standing waves. Furthermore, in [6], Morita and Ninomiya constructed two kinds of merging entire solutions. Very recently, Wang in [15] investigated the entire solutions for the degenerate Fisher equation by considering two traveling front solutions with critical speeds. And Zhang et al. in [16] dealt with the front-like entire solution of a classical nonlocal dispersal equation with ignition nonlinearity, where the dispersal kernel function may not be symmetric.

However, little is known about the entire solutions of reaction–diffusion equations with doubly degenerate nonlinearities. Thus, encouraged by [6, 12, 13, 15, 16], in this paper we will investigate the entire solutions of (1.1) under assumption $(A)$. Moreover, from [17] and the references therein, we know that the doubly degenerate reaction–diffusion equations usually mean that the diffusion term is assumed to be doubly degenerate. Here, from assumption $(A)$, we mainly focus on the reaction–diffusion equation with doubly degenerate nonlinearity.

The rest of this paper is arranged as follows. In Sect. 2, we show the existence and the asymptotic behaviors of traveling front solutions of (1.1) and give some preparations that will be used to construct the sup-sub solutions. An entire solution to (1.1) can be obtained by considering two traveling front solutions with critical speeds that come from both sides of the *x*-axis in Sect. 3. Finally, we apply our results to an example.

## Preliminaries

In this section, we will give some relevant preparations in order to obtain the main conclusions later. First of all, we state the definitions of supersolution and subsolution of (1.1) as follows.

### Definition 2.1

Set $\Omega:=\mathbb{R}\times[r, R]$ for some $R>r$. $\underline {u}(x,t)$ is called a subsolution of (1.1) in Ω if $\underline{u}(x,t)\leq u(x,t)\ ((x,t)\in\Omega)$ for every solution of (1.1) defined in Ω such that $\underline{u}(x,r)\leq u(x,r)$ $(x\in\mathbb{R})$. If $\underline{u}(x,t)$ is a subsolution of (1.1) in $\mathbb {R}\times[r,-T]$ for any $r<-T$, then $\underline{u}(x,t)$ is called a subsolution of (1.1) in $\mathbb{R}\times[-\infty,-T]$ for some $T\geq0$. Similarly, we can define a supersolution by reversing the inequalities.

Let $\mathcal{F}(u)=u_{t}-u_{xx}-f(u)$. We notice that a bounded function $u(x,t)$ is a subsolution of (1.1) in $\mathbb{R}\times(-\infty , -T)\ (T\geq0)$ if $\mathcal{F}(u)\leq0 \mbox{ for } (x,t)\in\mathbb{R}\times(-\infty, -T)$, while it is a supersolution if $\mathcal{F}(u)\geq0$.

From [3], we can obtain the following result, which shows the existence and the asymptotic behavior of traveling front solutions of (1.1).

### Lemma 2.2

([3])

*Suppose that* $(A)$ *holds*, *then there is a unique positive number* $c^{*}$ *such that*, *for any* $c\geq c^{*}$, (1.2) *has a monotonically increasing solution* $\phi(z)$, *while for* $c^{*}>c>0$, *there is no positive solution for* (1.2). *Moreover*, *the solutions of* (1.2) *have the following asymptotic behaviors*:

2.1 $$\begin{aligned} \phi(z)= \textstyle\begin{cases} Be^{c^{*}z}+o(e^{c^{*}z})& \textit{as }z\rightarrow-\infty, \textit{ if }c=c^{*},\\ H_{0} (\frac{1}{c}z(1+o(1)) )& \textit{as }z\rightarrow-\infty,\textit{ if }c>c^{*},\\ H_{1} (\frac{1}{c}z(1+o(1)) )& \textit{as }z\rightarrow+\infty,\textit{ if }c\geq c^{*}, \end{cases}\displaystyle \end{aligned}$$

*where* $B>0$ *is a constant*, $H_{0}=F_{0}^{-1}, F_{0}=\int_{u_{0}}^{u}\frac{{\mathrm {d}}s}{f(s)},0< u< u_{0}$ *for some small* $u_{0}$, $H_{1}=F_{1}^{-1},F_{1}=\int _{u_{1}}^{u}\frac{{\mathrm {d}}s}{f(s)},u_{1}< u<1$ *and* $u_{1}$ *is close to* 1.

By Lemma 2.2, it is obvious that there are positive constants $r_{i}$ ($i=1,2$), *α* and *η* such that

2.2 $$\begin{aligned} &r_{1}e^{\alpha z}\leq\phi(z)\leq r_{2}e^{\alpha z},\quad z \leq0, \end{aligned}$$

2.3 $$\begin{aligned} &\inf_{z\leq0}\frac{\phi'(z)}{\phi(z)}=\eta, \end{aligned}$$

where $\alpha\leq c^{*}$ and $\phi(z)$ is the traveling front solution with critical speed. For simplicity of statements, we denote *c* as $c^{*}$ in the following.

In order to establish the supersolution of (1.1), we should consider the following ordinary differential problem:

2.4 $$\begin{aligned} \textstyle\begin{cases} p'(t)=c+Me^{\alpha p},\quad t< 0,\\ p(0)< 0, \end{cases}\displaystyle \end{aligned}$$

where

2.5 $$\begin{aligned} M>\max \biggl\{ \frac{Lr_{2}^{2}}{2\eta r_{1}}, \frac{Lr_{2}}{\eta} \biggr\} \end{aligned}$$

and $L:=\max_{u\in[0,2]} \vert f''(u) \vert $. From direct calculation, we can obtain the solution of (2.4) explicitly as

$$p(t)=ct-\frac{1}{\alpha}\ln \biggl\{ e^{-\alpha p(0)}+\frac{M(1-e^{c\alpha t})}{c} \biggr\} < 0,\quad t\leq0, $$

and

$$q=\lim_{t\rightarrow-\infty} \bigl(p(t)-ct \bigr)=-\frac{1}{\alpha}\ln \biggl\{ e^{-\alpha p(0)}+\frac{M}{c} \biggr\} . $$

Moreover, there exists $K>0$ so that for $t\leq0$

$$0< p(t)-ct-q\leq Ke^{c\alpha t}. $$

## The existence of entire solutions

In this section, we discuss the existence of entire solutions of (1.1). Firstly, we construct the supersolution.

### Lemma 3.1

*Assume that* $(A)$ *holds*, *and let* $p(t)$ *be the solution of* (2.4). *Then the function*

$$\bar{u}(x,t)=\phi \bigl(x+p(t) \bigr)+\tilde{\phi} \bigl(-x+p(t) \bigr) $$

*is a supersolution of* (1.1) *for* $t\leq0$, *where* $\tilde{\phi}(-x+p(t))=\phi(-x+p(t))$.

### Proof

For simplicity, we use *ϕ* and *ϕ̃* instead of $\phi (x+p(t))$ and $\tilde{\phi}(-x+p(t))$. Then we have

3.1 $$\begin{aligned} \mathcal{F}(\bar{u})&=\phi'p'+ \tilde{\phi}'p'-\phi ''- \tilde{\phi}''-f(\phi+\tilde{\phi}) \\ &= \bigl(\phi'+\tilde{\phi}' \bigr) \bigl(p'-c \bigr)-f(\phi+\tilde{\phi})+f(\phi)+f(\tilde{\phi }) \\ &= \bigl(\phi'+\tilde{\phi}' \bigr) \bigl[Me^{\alpha p}-G(x,t) \bigr], \end{aligned}$$

where

$$G(x,t):=\frac{f(\phi+\tilde{\phi})-f(\phi)-f(\tilde{\phi})}{\phi'+\tilde {\phi}'}. $$

Now we need to divide $\mathbb{R}$ into three parts to estimate $G(x,t)$.

(i) For $p\leq x\leq-p$, then $x+p\leq0, -x+p\leq0$. Noting that $f(0)=0,f\in C^{2}([0,2])$ and $\phi,\tilde{\phi}\in[0,1]$, we can get

$$f(\phi+\tilde{\phi})-f(\phi)-f(\tilde{\phi})= \biggl( \int_{0}^{1}f'(\phi +s\tilde{\phi})\,{ \mathrm {d}}s \biggr)\tilde{\phi}- \biggl( \int_{0}^{1}f'(s\tilde{\phi})\,{\mathrm {d}}s \biggr)\tilde{\phi}\leq L\phi \tilde{\phi}. $$

Combining with (2.2) and (2.3), it follows that

3.2 $$\begin{aligned} G(x,t)\leq\frac{L\phi\tilde{\phi}}{\eta(\phi+\tilde{\phi})}\leq\frac {Lr_{2}^{2}e^{2\alpha p}}{2\eta r_{1}e^{\alpha p}}=\frac{Lr_{2}^{2}}{2\eta r_{1}}e^{\alpha p}. \end{aligned}$$

(ii) For $x\leq p$, then $x+p\leq0, -x+p\geq0$. Now we can modify $f'(u)$ for $u\in(1,2]$ so that $f'(u)< f'(0)=0$ for $u\in(1,2)$. Noting hypothesis $(A)$, it follows that there exists $\delta\in(0,1)$ such that

3.3 $$\begin{aligned} f'(u)< f'(0),\quad u\in(1-\delta,2). \end{aligned}$$

Factually, (3.3) can be assured by translating $\phi(z)$ along *z*-axis. In the following, we may assume that

3.4 $$\begin{aligned} \phi(z)\geq1-\delta\quad\mbox{for any }z\geq0. \end{aligned}$$

Then it follows from (3.3) and (3.4) that

$$\begin{aligned}f(\phi+\tilde{\phi})-f(\phi)-f(\tilde{\phi})&= \biggl( \int _{0}^{1}f'(\tilde{\phi}+s\phi)\,{ \mathrm {d}}s \biggr)\phi - \biggl( \int_{0}^{1}f'(s\phi)\,{\mathrm {d}}s \biggr)\phi \\ &\leq\phi \int_{0}^{1} \bigl\vert f'(0)-f'(s \phi) \bigr\vert \,{\mathrm {d}}s\leq L\phi^{2}. \end{aligned} $$

Then

3.5 $$\begin{aligned} G(x,t)\leq\frac{L\phi^{2}}{\phi'+\tilde{\phi}'}\leq\frac{L\phi}{\phi'/\phi }\leq\frac{Lr_{2}e^{\alpha(x+p)}}{\eta}\leq \frac{Lr_{2}}{\eta}e^{\alpha p}. \end{aligned}$$

(iii) For $x\geq-p$, then $x+p\geq0, -x+p\leq0$. Noting that $G(x,t)=G(-x,t)$ and (3.5), we get

3.6 $$\begin{aligned} G(x,t)\leq\frac{Lr_{2}}{\eta}e^{\alpha p}. \end{aligned}$$

Then, combining (2.5), (3.1), (3.2), (3.5), and (3.6), we have

$$\mathcal{F}(\bar{u})= \bigl(\phi'+\tilde{\phi}' \bigr) \bigl[Me^{\alpha p}-G(x,t) \bigr]\geq0. $$

The proof is complete. □

### Theorem 3.1

*Assume that* $(A)$ *holds*. *Let* *ϕ* *and* *ϕ̃* *be traveling front solutions of* (1.1) *with the critical speed* *c*. *Then*, *for arbitrarily given constants* $\theta_{1}, \theta_{2}$, *there exists an entire solution* $u(x,t)$ *which satisfies*

3.7 $$\begin{aligned} &\lim_{t\rightarrow-\infty} \Bigl\{ \sup_{x\geq0} \bigl\vert u(x,t)-\phi(x+ct+\theta _{1}) \bigr\vert +\sup_{x\leq0} \bigl\vert u(x,t)-\tilde{\phi}(-x+ct+\theta_{2}) \bigr\vert \Bigr\} =0, \end{aligned}$$

3.8 $$\begin{aligned} &\lim_{t\rightarrow+\infty}\sup_{x\in\mathbb{R}} \bigl\vert u(x,t)-1 \bigr\vert =0. \end{aligned}$$

*Moreover*, *if* $c\alpha>N=\max_{u\in[0,2]} \vert f'(u) \vert $, *then this entire solution is unique*. *Furthermore*, *this solution satisfies*

(i) $\frac{\partial u(x,t)}{\partial t}>0$ *for all* $(x,t)\in\mathbb{R}\times\mathbb{R}$;
(ii) $\lim_{t\rightarrow-\infty}\sup_{x\in [\alpha, \beta]} \vert u(x,t) \vert =0$ *with* $\alpha, \beta\in\mathbb{R}$ *and* $\alpha<\beta$;
(iii) $\lim_{ \vert x \vert \rightarrow\infty}\sup_{t\in[S, \infty )} \vert u(x,t)-1 \vert =0 $ *with* $S\in\mathbb{R}$.

### Proof

Define

$$\underline{u}(x,t)=\max \bigl\{ \phi(x+ct+q),\tilde{\phi}(-x+ct+q) \bigr\} . $$

It is obvious that

$$\underline{u}(x,t)\leq\bar{u}(x,t),\quad (x,t)\in\mathbb{R}\times(-\infty,0]. $$

Combining with the comparison principle, it yields that there exists a solution $\tilde{u}(x,t)$ of (1.1) which satisfies

$$\underline{u}(x,t)\leq\tilde{u}(x,t)\leq\bar{u}(x,t),\quad\mbox{for }(x,t)\in \mathbb{R}\times(-\infty,0]. $$

Consider the following problem:

3.9 $$\begin{aligned} \textstyle\begin{cases} u_{t}=u_{xx}+f(u),\quad (x,t)\in\mathbb{R}\times[0,+\infty),\\ u(x,0)=\tilde{u}(x,0),\quad x\in\mathbb{R}. \end{cases}\displaystyle \end{aligned}$$

It is easy to see that there is a unique solution $u(x,t)$ of (3.9) which also satisfies $\underline{u}(x,t)\leq u(x,t)\leq1$ for any $(x,t)\in\mathbb{R}\times[0,+\infty)$. Let

$$u(x,t)=\tilde{u}(x,t)\quad\mbox{for } (x,t)\in\mathbb{R}\times(-\infty,0]. $$

Then we can obtain an entire solution of (1.1) which satisfies

$$\underline{u}(x,t)\leq u(x,t)\leq\bar{u}(x,t)\quad\mbox{for }(x,t)\in \mathbb{R} \times(-\infty,0] $$

and

$$\underline{u}(x,t)\leq u(x,t)\leq1\quad\mbox{for }(x,t)\in\mathbb{R}^{2}. $$

Next we will prove (3.7). Firstly, we prove

3.10 $$\begin{aligned} \lim_{t\rightarrow-\infty} \Bigl\{ \sup_{x\geq0} \bigl\vert u(x,t)-\phi(x+ct+q) \bigr\vert +\sup_{x\leq0} \bigl\vert u(x,t)-\tilde{\phi}(-x+ct+q) \bigr\vert \Bigr\} =0. \end{aligned}$$

Then, for $x\geq0$ and $t\leq0$, we can get

$$\begin{aligned}0\leq u(x,t)-\phi(x+ct+q)&\leq\bar{u}(x,t)- \phi(x+ct+q) \\ &\leq\phi \bigl(x+p(t) \bigr)+\tilde{\phi} \bigl(-x+p(t) \bigr)-\phi(x+ct+q) \\ &\leq r_{2}e^{\alpha(-x+p(t))}+\sup_{z\in\mathbb{R}} \bigl\vert \phi '(z) \bigr\vert \bigl(p(t)-ct-q \bigr) \\ &\leq r_{2}e^{\alpha p(t)}+K\sup_{z\in\mathbb{R}} \bigl\vert \phi'(z) \bigr\vert e^{c\alpha t}. \end{aligned} $$

For $x\leq0$ and $t\leq0$, similarly, we obtain

$$0\leq u(x,t)-\tilde{\phi}(-x+ct+q)\leq r_{2}e^{\alpha p(t)}+K\sup _{z\in \mathbb{R}} \bigl\vert \tilde{\phi}'(z) \bigr\vert e^{c\alpha t}. $$

For any given $\theta_{1}, \theta_{2}$, by choosing

$$\mu=\frac{\theta_{1}-\theta_{2}}{2},\qquad \tau=\frac{\theta_{1}+\theta_{2}-2q}{2c}, $$

we can obtain the entire solution of (1.1) as $\hat {u}(x,t)=u(x+\mu, t+\tau)$.

Now we discuss the uniqueness. Set $u_{1}(x,t), u_{2}(x,t)$ be two entire solutions of (1.1). Suppose that $u_{1}(x,t)\geq u_{2}(x,t)$. Noting that $\max\{u_{1}(x,t), u_{2}(x,t)\}$ is a subsolution, there exists an entire solution $\hat{u}(x,t)$ satisfying

$$\max \bigl\{ u_{1}(x,t), u_{2}(x,t) \bigr\} \leq\hat{u}(x,t) \leq \bar{u}(x,t)\quad (t\leq0). $$

For any given $(x,t),t\leq0$, let $\tau\leq t$. Setting $v(x,t):=u_{1}(x,t)-u_{2}(x,t)$, we have

$$v_{t}=v_{xx}+ \biggl( \int_{0}^{1}f' \bigl(u_{2}+s(u_{1}-u_{2}) \bigr)\,{\mathrm {d}}s \biggr)v\leq v_{xx}+Nv. $$

Then

$$\begin{aligned}v(x,t)&\leq\frac{e^{N(t-\tau)}}{\sqrt{4\pi(t-\tau)}} \int _{-\infty}^{\infty}e^{-\frac{(x-y)^{2}}{4(t-\tau)}}v(y,\tau)\,{\mathrm {d}}y \\ &=\frac{e^{N(t-\tau)}}{\sqrt{\pi}} \int_{-\infty}^{\infty}e^{-z^{2}}v(x-2\sqrt{t-\tau}z, \tau)\,{\mathrm {d}}z. \end{aligned} $$

Furthermore, from the above estimates it also follows that there exists $D>0$ such that

$$v(x,t)\leq\bar{u}(x,t)-\max \bigl\{ \phi(x+ct+q), \tilde{\phi}(-x+ct+q) \bigr\} \leq De^{c\alpha t}\quad (t\leq0). $$

Thus we can get

$$0\leq v(x,t)\leq De^{Nt}e^{(c\alpha-N)\tau}. $$

Since *τ* can be made arbitrarily small and $c\alpha>N$, we can obtain that $v(x,t)=0$ for $x\in\mathbb{R}$. This completes the proof. □

Next we give another type of entire solutions. Suppose that $\zeta(t)$ is a solution of the following ordinary differential equation:

3.11 $$\begin{aligned} \dot{\zeta}=f(\zeta)+N\zeta, \end{aligned}$$

where *N* is defined in Theorem 3.1 and $0<\zeta(t)<1$.

### Theorem 3.2

*Assume that* (*A*) *holds*. *Define* $\rho(t):=\rho_{0}e^{Nt}$, *and let* $\rho_{0}>0$ *satisfy*

$$0< \zeta(t)-\rho(t)\leq R_{0}e^{Nt}\quad (t\leq0), $$

*where* $R_{0}$ *is a positive number*. *For given* $c_{1}, c_{2}\geq c^{*},\nu_{1}, \nu_{2}\in\mathbb{R}$ *and positive constants* $k_{j}\ (j=1,2),R_{1}$ *and* *T*, *there exist monotone increasing functions* $p_{j}(t)\ (j=1,2)$ *satisfying*

$$\bigl\vert p_{j}(t)-c_{j}t-\nu_{j} \bigr\vert \leq R_{1}e^{k_{j}t}\quad(t\leq-T) $$

*such that* $\bar{u}_{ik}(x,t):=\chi_{i}\phi(x+p(t))+\chi_{k}\tilde{\phi }(-x+p(t))+\rho(t)\ ((i,k)=(1,0),(0,1),(1,1))$ *are supersolutions for* $t\in(-\infty, -T]$, *where* $\chi_{i}=i\ (i=0,1)$. *Furthermore*, *for* $(x,t)\in\mathbb{R}\times (-\infty,-T]$, *there are entire solutions* $u_{ik}(x,t),\ (i,k)=(1,0),(0,1),(1,1)$ *of* (1.1) *which satisfy*

3.12 $$\begin{aligned} \max \bigl\{ \chi_{i}\phi(x+ct+\theta_{1}), \chi_{k}\tilde{\phi}(-x+ct+\theta_{2}), \zeta(t) \bigr\} \leq u_{ik}(x,t)\leq\min \bigl\{ \bar{u}_{ik}(x,t), 1 \bigr\} . \end{aligned}$$

### Proof

We just need to prove that $\bar{u}_{ik}(x,t)$ is a supersolution of (1.1). Now set

$$\bar{u}(x,t)=\phi \bigl(x+p(t) \bigr)+\tilde{\phi} \bigl(-x+p(t) \bigr)+\rho(t). $$

Similarly, we can have that

$$\begin{aligned}\mathcal{F}(\bar{u})&=\phi'p'+ \tilde{\phi}'p'+\rho'-\phi ''-\tilde{\phi}''-f(\phi+ \tilde{\phi}+\rho) \\ &= \bigl(\phi'+\tilde{\phi}' \bigr) \bigl(p'-c \bigr)-f(\phi+\tilde{\phi}+\rho)+f(\phi)+f(\tilde {\phi})+ \rho' \\ &= \bigl(\phi'+\tilde{\phi}' \bigr)Me^{\alpha p}-H(x,t)+ \rho'(t), \end{aligned} $$

where

$$H(x,t)=f(\phi+\tilde{\phi}+\rho)-f(\phi)-f(\tilde{\phi}). $$

If $x\leq0$, we can get

$$\begin{aligned}H&=f(\phi+\tilde{\phi}+\rho)-f(\tilde{\phi}+\rho)-f( \phi )+f(\tilde{\phi}+\rho)-f(\tilde{\phi}) \\ &= \biggl( \int_{0}^{1}f' (\tilde{\phi}+\rho+s\phi )\,{\mathrm {d}}s \biggr)\phi- \biggl( \int_{0}^{1}f'(s\phi)\,{\mathrm {d}}s \biggr)\phi+ \biggl( \int_{0}^{1}f' (\tilde{\phi}+s\rho )\,{ \mathrm {d}}s \biggr)\rho \\ &\leq \biggl( \int_{0}^{1} \bigl[f'(0)-f'(s \phi) \bigr]\,{\mathrm {d}}s \biggr)\phi+N\rho \\ &\leq L\phi^{2}+N\rho. \end{aligned} $$

Noting that $\rho'=N\rho$ and

$$\frac{L\phi^{2}}{\phi'+\tilde{\phi}'}\leq\frac{Lr_{2}}{\eta}e^{\alpha p}, $$

then it follows that

$$\mathcal{F}(\bar{u})\geq \bigl(\phi'+\tilde{\phi}' \bigr) \bigl[Me^{\alpha p}-(Lr_{2}/\eta )e^{\alpha p} \bigr] \geq0. $$

Similarly, we can prove that $\mathcal{F}(\bar{u})\geq0$ if $x\geq0$. That is, $\bar{u}(x,t)$ is a supersolution of (1.1). The rest of the proof is similar to that of [8]. □

## Applications

For the double degenerated generalized Fisher-type equation,

4.1 $$\begin{aligned} u_{t}=u_{xx}+u^{p}(1-u)^{q},\quad p>1,q>1, \end{aligned}$$

where *p* and *q* are not necessarily integers. Now we show the conclusion of the existence of traveling wave front solutions of (4.1).

### Lemma 4.1

([4])

*If* $p>1,q>1$, *there exists* $c^{*}(p,q)>0$ *such that*, *for any* $c\geq c^{*}(p,q)$, *there are traveling front solutions* $\phi_{c}(z)\ (z=x-ct)$ *connecting* $u=0$ *and* $u=1$ *which satisfy*

$$\begin{aligned} &\phi(z)\sim \textstyle\begin{cases}e^{-c^{*}z}& \textit{as }z\rightarrow+\infty,\textit{ if }c=c^{*}(p,q),\\ [\frac{c}{(p-1)z} ]^{\frac{1}{p-1}}& \textit{as }z\rightarrow +\infty,\textit{ if }c>c^{*}(p,q), \end{cases}\displaystyle \\ &1-\phi_{c}(z)\sim \biggl[\frac{c}{(q-1) \vert z \vert } \biggr]^{\frac{1}{q-1}},\quad \textit{as }z\rightarrow-\infty, \textit{ if }c\geq c^{*}(p,q). \end{aligned}$$

It is easy to verify that $f(u)=u^{p}(1-u)^{q}$ satisfy assumption $(A)$ in Theorem 3.1. Thus we have the following result for (4.1).

### Theorem 4.1

*Assume that* $p>1, q>1$. *Let* *ϕ* *and* *ϕ̃* *be traveling front solutions of* (4.1) *with the minimum speed* *c*. *Then*, *for arbitrarily given constants* $\theta_{1}, \theta_{2}$, *there exists an entire solution* $u(x,t)$ *which satisfies*

$$\begin{aligned} &\lim_{t\rightarrow-\infty} \Bigl\{ \sup_{x\geq0} \bigl\vert u(x,t)-\phi(x+ct+\theta _{1}) \bigr\vert +\sup_{x\leq0} \bigl\vert u(x,t)-\tilde{\phi}(-x+ct+\theta_{2}) \bigr\vert \Bigr\} =0, \\ &\lim_{t\rightarrow+\infty}\sup_{x\in\mathbb{R}} \bigl\vert u(x,t)-1 \bigr\vert =0. \end{aligned}$$
